# The Functional Severity Assessment of Coronary Stenosis Using Coronary Computed Tomography Angiography-Based Myocardial Mass at Risk and Minimal Lumen Diameter

**DOI:** 10.1155/2020/6716130

**Published:** 2020-01-30

**Authors:** Kenji Sadamatsu, Kazuhiro Nagaoka, Yasuaki Koga, Kotaro Kagiyama, Kohei Muramatsu, Kiyoshi Hironaga, Hideki Tashiro, Takafumi Ueno, Yoshihiro Fukumoto

**Affiliations:** ^1^Department of Cardiology, St. Mary's Hospital, Kurume, Japan; ^2^Department of Cardiovascular Medicine, Fukuoka City Hospital, Fukuoka, Japan; ^3^Department of Cardiovascular Medicine, Oita Prefectural Hospital, Oita, Japan; ^4^Division of Cardiovascular Medicine, Department of Internal Medicine, Kurume University School of Medicine, Kurume, Japan

## Abstract

**Background:**

We investigated whether or not the addition of myocardial mass at risk (MMAR) to quantitative coronary angiography was useful for diagnosing functionally significant coronary stenosis in the daily practice.

**Methods:**

We retrospectively enrolled 111 consecutive patients with 149 lesions who underwent clinically indicated coronary computed tomography angiography and subsequent elective coronary angiography with fractional flow reserve (FFR) measurement. MMAR was calculated using a workstation-based software program with ordinary thin slice images acquired for the computed tomography, and the minimal lumen diameter (MLD) and the diameter stenosis were measured with quantitative coronary angiography.

**Results:**

The MLD and MMAR were significantly correlated with the FFR, and the MMAR-to-MLD ratio (MMAR/MLD) showed a good correlation. The area under the receiver operating characteristic curve (AUC) of MMAR/MLD for FFR ≤ 0.8 was 0.746, and the sensitivity, specificity, positive predictive value, and negative predictive value were 60%, 83%, 68%, and 77%, respectively, at a cut-off value of 29.5 ml/mm. The addition of MMAR/MLD to diameter stenosis thus made it possible to further discriminate lesions with FFR ≤ 0.8 (AUC = 0.750). For the proximal left coronary artery lesions, in particular, MMAR/MLD showed a better correlation with the FFR, and the AUC of MMAR/MLD for FFR ≤ 0.8 was 0.919 at a cut-off value of 31.7 ml/mm.

**Conclusions:**

The index of MMAR/MLD correlated well with the physiological severity of coronary stenosis and showed good accuracy for detecting functional significance. The MMAR/MLD might be a useful parameter to consider when deciding the indication for revascularization.

## 1. Introduction

The fractional flow reserve (FFR) is now considered the reference for the evaluation of myocardial ischemia and the expected benefit from revascularization of coronary stenosis [1–3]. Several randomized clinical trials have clearly demonstrated the superiority of FFR-guided coronary intervention to angiography-guided coronary intervention in terms of an improved clinical outcome and saving medical costs, so FFR measurement plays an integral role in the management of patients with coronary artery disease [4]. However, a physiological examination is not practical in all patients in daily practice, as it is an invasive procedure that needs a dedicated wire with an extra cost, although the complication rate is deemed quite low [5].

Several hypotheses have been proposed for the discordance between anatomical stenosis and functional severity. As is already well known, ischemia is determined not only by anatomical stenosis but also by the amount of myocardial mass distal to the culprit lesion [6]. A lesion subtended by a large myocardial mass may be associated with a large increase in coronary blood flow during hyperemia and result in a larger decrease in the FFR in comparison to a lesion subtended by a small myocardial mass, despite the similar degree of anatomical stenosis. Thus, the myocardial mass at risk (MMAR), representing the volume of myocardium distal to the culprit lesion, might be an important factor in determining the functional severity of a culprit lesion and for explaining this discordance. Actually, several reports have demonstrated a correlation between the myocardial supply area distal to the site of stenosis and the FFR [6–8], and a few recent studies have shown that the MMAR improved the accuracy of using anatomical information to predict functional stenosis with a novel system based on the Voronoi tessellation or allometric scaling law [9, 10]. We have been able to measure the MMAR easily with only a few clicks using commercially available software programs.

Given the above, we investigated whether or not the addition of the MMAR to quantitative coronary angiography (QCA) was useful for diagnosing functionally significant stenosis in daily practice.

## 2. Materials and Methods

### 2.1. Study Design

**Figure 1:**
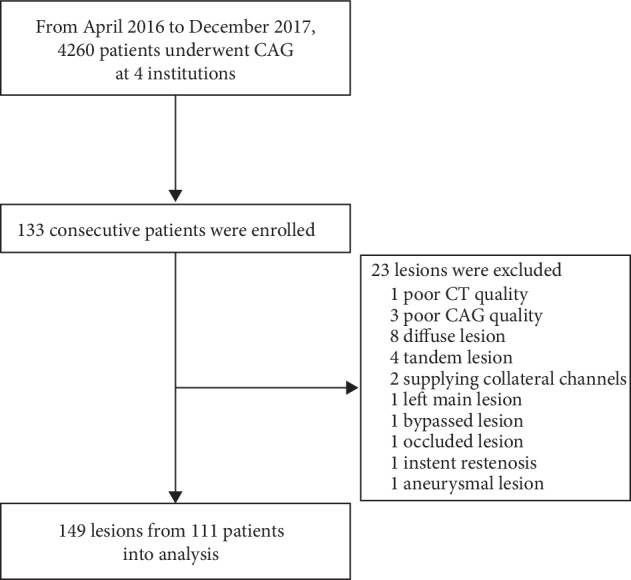
Study enrollment. CAG, coronary angiography; CT, computed tomography.

This study was conducted at four sites (St. Mary's Hospital, Kurume, Japan; Fukuoka City Hospital, Fukuoka, Japan; Kurume University Hospital, Kurume, Japan; Oita Prefectural Hospital, Oita, Japan). From April 2016 to December 2017, we retrospectively enrolled 133 consecutive patients who underwent clinically indicated coronary computed tomography angiography (CCTA) and subsequent elective coronary angiography (CAG) with a physiological assessment after the exclusion of patients with ST-segment elevation myocardial infarction, uncompensated heart failure, bypass surgery, complex structural or congenital heart disease, or any clinical instability or life-threatening disease. Coronary vessels with significant diffuse lesions, tandem lesions, left main lesions, and collateral channels supplying occluded lesions were then excluded [11]. Coronary lesions that were not clearly opacified on CAG were also excluded. Ultimately, 111 patients with 149 lesions were collected for the analysis (Figure 1).

All patients gave informed consent for the procedure. The study protocol conforms to the ethical guidelines of the 1975 Declaration of Helsinki as reflected in a priori approval by the institution's human research committee at each center.

### 2.2. FFR and QCA

CAG and FFR measurements were performed in accordance with the standard protocol. In brief, at least 2 optimized projections were obtained for each major coronary artery after the administration of intracoronary nitroglycerin. FFR was measured using a pressure wire (PressureWire Certus or PressureWire Aeris, Abbott Vascular, Santa Clara, CA, USA; Verrata Pressure Guide Wire, Volcano, San Diego, CA, USA; OptoWire, Opsens Medical, Quebec, Canada) with the administration of intracoronary nicorandil [12, 13], papaverine, or intravenous adenosine [3]. QCA was performed with a computer-assisted automatic arterial contour detection system (CAAS, Pie Medical Imaging, Maastricht, The Netherlands) [14] by certified cardiologists who were blinded to the results of FFR and MMAR. Lesion length, diameter stenosis, and the minimal luminal diameter (MLD) were measured using end-diastolic angiographic images with an optimal projection showing minimal foreshortening of the lesion. Decisions to revascularize the lesions were made by the agreement of the attending physician and certified interventional cardiologists based on the results of the FFR measurements.

### 2.3. CCTA Image Acquisition

All CCTA images were obtained using multivendor CT scanners equipped with 64 or more detectors (Aquilion One, Canon Medical Systems, Otawara, Japan; Somatom definition AS64, Siemens, Forchheim, Germany; Revolution or Optima CT660, GE Healthcare, Chicago, IL, USA; iCT, Phillips, Amsterdam, The Netherlands) in accordance with the institutional standard protocol [15]. Automatic reconstruction was performed using the vendor-specific software program to determine the optimal phase of images.

### 2.4. Image Processing and Calculation of MMAR

**Figure 2:**
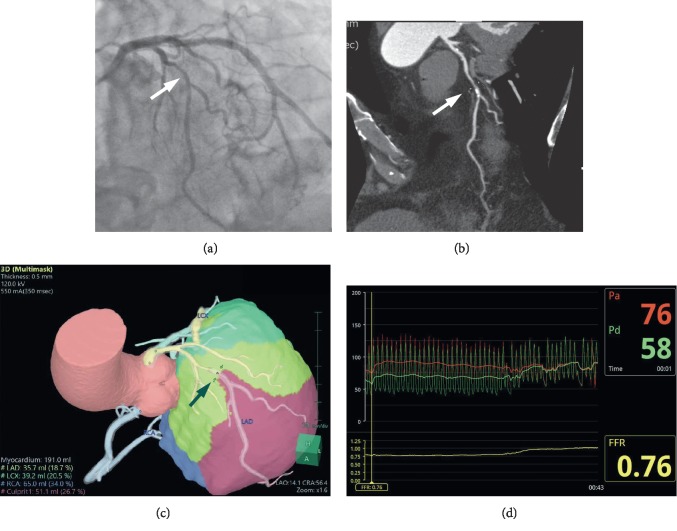
(a) Left coronary angiography (left cranial view) and (b) computed tomography angiography showed a calcified lesion (arrows) at the midsegment of the left anterior descending artery; the minimal lumen diameter (MLD) was 1.39 mm. (c) After setting a specific point at the culprit lesion (arrow), the software program (Synapse Vincent, Fujifilm Corp., Japan) showed that the myocardial mass at risk (MMAR) was 51.1 ml and that the %MMAR was 26.7%. Thus, the MMAR/MLD value was 36.8, which was highly predictive of fractional flow reserve (FFR) ≤0.8. (d) The invasive measurement revealed that the FFR was 0.76.

Standard DICOM data were transferred to a 3-dimensional (3D) imaging workstation incorporating the novel software program (Synapse Vincent, Fujifilm Corp, Tokyo, Japan). A 3D reconstruction of the coronary arteries and left ventricular myocardium was made after automatic and additional manual tracing of all visible coronary branches by an experienced technologist and confirmed by an experienced cardiologist who was blinded to the FFR values. The myocardial territory was calculated using an algorithm based on Voronoi tessellation, and the details have already been described [16, 17]. After setting a specific point at the culprit lesion in the coronary artery tree, the corresponding myocardial volume, i.e., MMAR, was calculated by including all voxels distal to the point and closer to the compromised coronary artery than any others. The %MMAR was then calculated automatically as the percentage of the corresponding myocardium in relation to the total left ventricular myocardial mass.  Figure 2 shows a representative case.

### 2.5. Statistical Analyses

**Figure 3:**
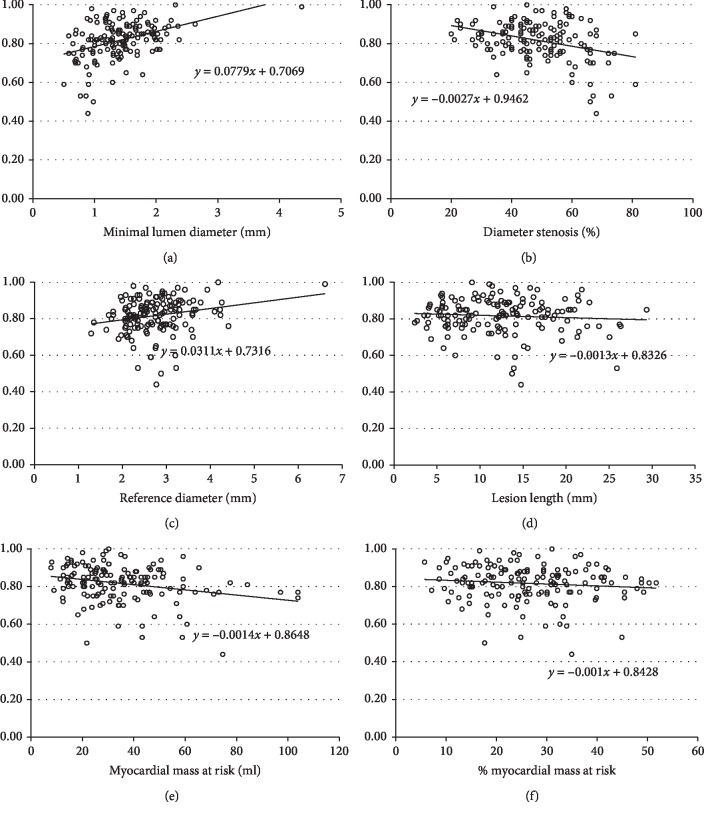
Correlations between the fractional flow reserve (FFR) and the parameters of quantitative coronary angiography or cardiac computed tomography. The relationships were significant for minimal lumen diameter (*r* = 0.40, *p* < 0.001; (a)), diameter stenosis (*r* = −0.35, *p* < 0.001; (b)), and reference diameter (*r* = 0.22, *p*=0.008; (c)) but not significant for lesion length (*p*=0.356; (d)) among the parameters of quantitative coronary angiography. Among the parameters of cardiac computed tomography, the myocardial mass at risk (MMAR) was significantly correlated with the FFR (*r* = −0.26, *p*=0.001; (e)), but the %MMAR was not (*p*=0.191; (f)).

Categorical variables are presented as frequencies and percentages. Continuous variables are shown as the median values with the first and third quartiles in parentheses. Differences between the 2 groups were evaluated using the chi-squared test for categorical variables and the Mann–Whitney *U* test for continuous variables. The FFR, QCA, and CCTA data were handled on a continuous scale. Correlations among variables were assessed by the Pearson method. In addition, MLD and MMAR are factors that determine the myocardial blood flow supply and demand, respectively, and they were well correlated with the FFR (Figure 3). We, therefore, examined the MMAR-to-MLD ratio (MMAR/MLD), which may represent the ratio of myocardial blood flow demand to supply [9]. The performance of discrimination for FFR ≤ 0.8 was investigated in a receiver operating characteristic analysis using the DeLong method, and the improvement of discrimination by the addition of MMAR/MLD to angiographic stenosis was assessed using reclassification analyses, including net reclassification improvement and integrated discrimination improvement. A 2-tailed *p* value of <0.05 was considered statistically significant. The statistical analyses were performed using the *R* 3.5.0 with the EZR package [18].

## 3. Results

The baseline characteristics of 111 patients with 149 lesions are summarized in Table 1, and their QCA and CCTA data are summarized in Table 2. The culprit lesions with FFR ≤ 0.8 showed a significantly larger diameter stenosis (*p*=0.002) and a significantly smaller minimal lumen diameter (*p* < 0.001) than the lesions with FFR >0.8. The MMAR was significantly larger in the culprit lesions with FFR ≤ 0.8 (*p*=0.032).

**Figure 4:**
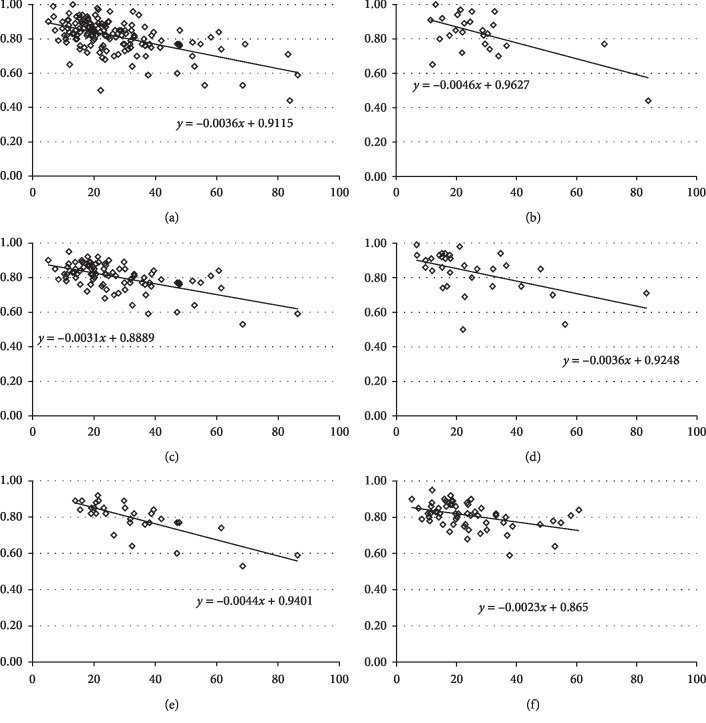
The myocardial mass at risk-to-minimal lumen diameter ratio (MMAR/MLD) was significantly correlated with the fractional flow reserve (FFR) (*r* = −0.56, *p* < 0.001; (a)). In the subgroup analysis, the relationships were also significant in the culprit lesions in the right coronary (*r* = −0.61, *p* < 0.001; (b)), left anterior descending (*r* = −0.59 *p* < 0.001; (c)), and left circumflex arteries (*r* = −0.51, *p*=0.003; (d)). In addition, significant relationships were noted in the culprit lesions in the proximal left anterior descending artery (*r* = −0.67, *p* < 0.001; (e)) and nonproximal left anterior descending artery (*r* = −0.43, *p* < 0.001; (f)).

A correlation analysis between the FFR and QCA parameters demonstrated that MLD (*r* = 0.40, *p* < 0.001; Figure 3(a)), diameter stenosis (*r* = −0.35, *p* < 0.001; Figure 3(b)), and reference diameter (*r* = 0.22, *p*=0.008; Figure 3(c)) had significant relationships with FFR, while lesion length did not (*p*=0.356; Figure 3(d)). With respect to CCTA, MMAR was significantly correlated with FFR (*r* = −0.26, *p*=0.001; Figure 3(e)), but %MMAR was not (*p*=0.191; Figure 3(f)). Intriguingly, the MMAR/MLD showed a good correlation with FFR (*r* = −0.56, *p* < 0.001; Figure 4(a)). In the subgroup analysis, a significant correlation with FFR was also noted in the culprit lesions in the right coronary (*r* = −0.61, *p* < 0.001; Figure 4(b)), the left anterior descending (*r* = −0.59, *p* < 0.001; Figure 4(c)), and the left circumflex arteries (*r* = −0.51, *p*=0.003; Figure 4(d)). In addition, the relationship was good in the culprit lesions in the proximal left anterior descending artery (*r* = −0.67, *p* < 0.001; Figure 4(e)), while a significant correlation was also noted in the culprit lesions in the nonproximal left anterior descending artery (*r* = −0.43, *p* < 0.001; Figure 4(f)). After excluding 4 lesions in the infarct-related vessels, the relationship remained good in the remaining 145 lesions (*r* = −0.53, *p* < 0.001).

**Figure 5:**
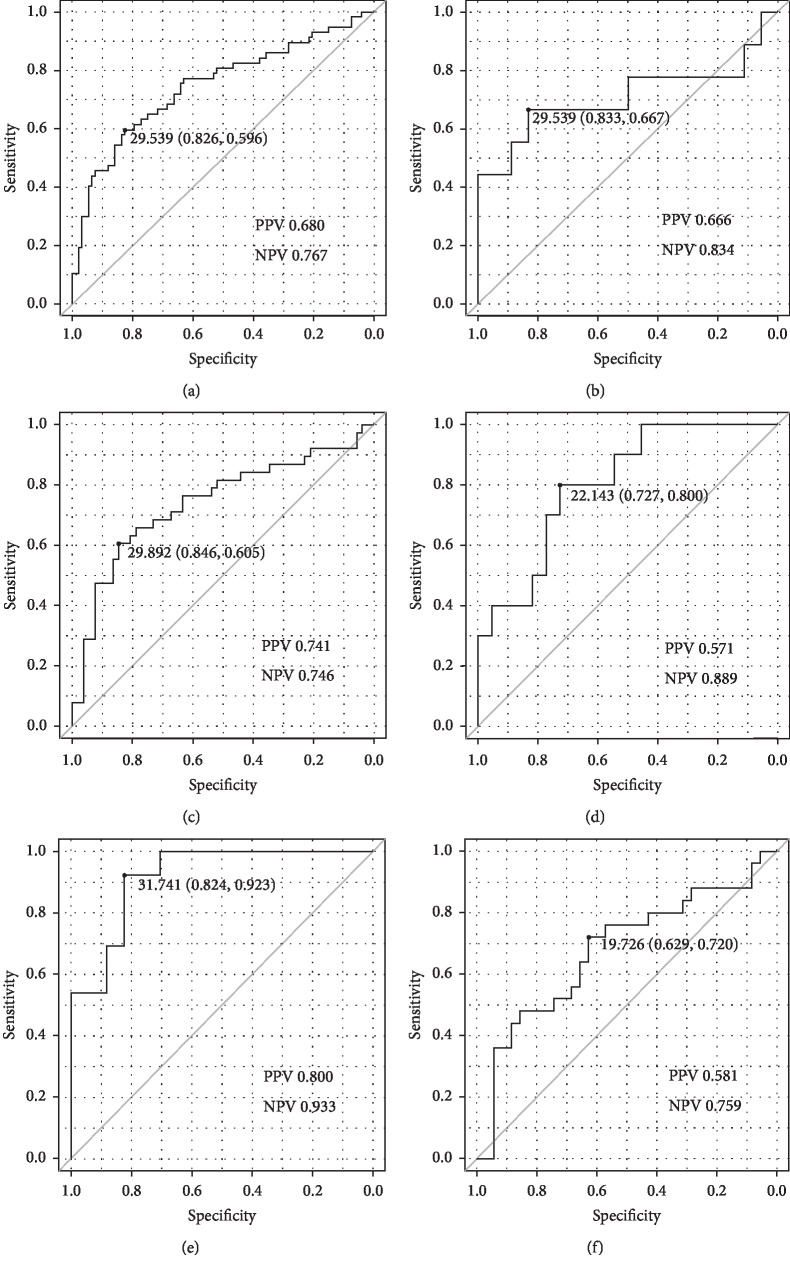
The areas under the receiver operating characteristic curve of myocardial mass at the risk-to-minimal lumen diameter ratio (MMAR/MLD) for FFR ≤ 0.8 in all culprit lesions (a) and in the culprit lesions in the right coronary (b), left anterior descending (c), and left circumflex arteries (d). In addition, the curves in the culprit lesions in the proximal (e) and nonproximal left anterior descending arteries (f). Each graph shows the optimum cut-off value (specificity, sensitivity). PPV: positive predictive value; NPV: negative predict value.

We, therefore, examined the diagnostic performance of discrimination for FFR ≤ 0.8. The area under the receiver operating characteristic curve (AUC) of MMAR/MLD for FFR ≤ 0.8 was 0.746 (95% confidence interval (CI): 0.6619–0.832; Figure 5(a)). The sensitivity, specificity, positive predictive value, and negative predictive value were 60%, 83%, 68%, and 77%, respectively, at a cut-off value of 29.5 ml/mm. In the subgroup analysis, the AUC of MMAR/MLD for FFR ≤ 0.8 in the right coronary, the left anterior descending, and the left circumflex arteries were 0.710 (95% CI: 0.447–0.972; Figure 5(b)), 0.741 (95% CI: 0.631–0.851; Figure 5(c)), and 0.805 (95% CI: 0.647–0.962; Figure 5(d)), respectively. In addition, the AUCs of MMAR/MLD for FFR ≤ 0.8 in the proximal and nonproximal left anterior descending arteries were 0.919 (95% CI: 0.824–1.000; Figure 5(e)) and 0.678 (95% CI: 0.533–0.822; Figure 5(f)). The cut-off values, specificity, sensitivity, positive predictive value, and negative predictive value are shown in Figure 5.

**Figure 6:**
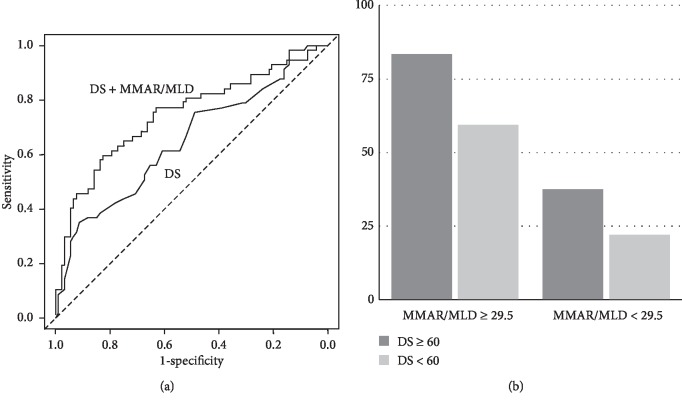
(a) The areas under the receiver operating characteristic curve for FFR ≤ 0.8 by diameter stenosis (DS) and by DS and myocardial mass at risk-to-minimal lumen diameter ratio (MMAR/MLD). (b) The percentage of FFR ≤ 0.8 in the culprit lesions according to MMAR/MLD 29.5 ml/mm and DS 60%.

Next, we examined whether the discrimination of FFR ≤ 0.8 was improved by MMAR/MLD in comparison to the conventional QCA parameter of diameter stenosis. The AUC of diameter stenosis for FFR ≤ 0.8 was 0.651 (95% CI: 0.558–0.744; Figure 6). The sensitivity, specificity, positive predictive value, and negative predictive value were 35%, 91%, 71%, and 69%, respectively, at a cut-off value of 60%. Then, the addition of MMAR/MLD to diameter stenosis further discriminated lesions with an FFR value of ≤0.8 (AUC, 0.750 [95% CI: 0.664–0.836]; net reclassification improvement, 0.544 [95% CI: 0.228–0.860], *p* < 0.001; integrated discrimination improvement, 0.131 [95% CI: 0.073–0.189], *p* < 0.001; Figure 6).

## 4. Discussion

In this study, we validated the diagnostic accuracy of a novel index that is easily obtained from QCA and CCTA data using a standard 3D analysis software program in order to predict functionally significant coronary artery stenosis. We found that the MMAR/MLD correlated well with FFR compared to ordinary QCA parameters, and a cut-off value of 29.5 ml/mm was predictive for FFR ≤ 0.8. The addition of MMAR/MLD to diameter stenosis further discriminated lesions with an FFR value of ≤0.8. The usefulness of the MMAR/MLD was consistent, regardless of the lesion location. For the proximal left coronary artery lesions, in particular, MMAR/MLD showed a good correlation with the FFR and a high diagnostic performance of discrimination for FFR ≤ 0.8 at a cut-off value of 31.7 ml/mm.

The percentage of myocardial ischemia on stress myocardial perfusion single-photon emission computed tomography has been proposed as an important factor for determining indications for revascularization, as it is associated with the prognosis of patients with coronary artery disease [19]. A good correlation between MMAR and the comprehensive summed stress score/summed difference score assessed with single-photon emission computed tomography has been shown [20]. Thus, the MMAR can also be expected to be a useful factor and might be superior because the MMAR only indicates the area perfused by the target lesions. However, the MMAR measurement has not been used widely. Angiographic scores were developed to assess the MMAR and provide prognostic information based on anatomical findings [21]; however, the routine application of this method in daily practice might be bothersome. Recently, a novel measurement system was developed, validated, and released [16, 17], and we are able to calculate the MMAR in only a few clicks using a software program with ordinary thin slice images acquired for CCTA.

Several estimation methods for MMAR have been reported for predicting functional coronary stenosis. The 3 scoring systems for evaluating MMAR were validated, and each score was found to be significantly correlated with FFR in patients with intermediate coronary stenosis [6]. Furthermore, the ratio of the Duke Jeopardy Score, one of the scoring systems for MMAR, and the MLD showed significantly high correlation with FFR (*r*^2^ = 0.59, *p* < 0.001) and demonstrated good diagnostic performance for predicting a positive or negative FFR value, with overall accuracy, sensitivity, specificity, positive predictive value, and negative predictive value of 86%, 94%, 82%, 71%, and 97%, respectively [22]. Thus, this index, which is similar to MMAR/MLD, seems promising. However, such scoring systems are not applicable in all patient subsets, being inappropriate in those with aberrant vessel anatomy, total occlusion, or damaged and thin myocardium, such as in cases of old myocardial infarction, because the estimated perfusion territories would be inaccurate.

A few recent studies have investigated the relationship between FFR and the MMAR calculated from CCTA images. Kang et al. evaluated the myocardial volume subtended by a stenotic coronary segment (which we refer to as MMAR), with the Voronoi algorithm and quantitative intravascular ultrasound data to assess functional coronary stenosis. The ratio of MMAR to minimal lumen area^2^ >4.04 predicted an FFR < 0.80 with sensitivity, specificity, positive predictive value, negative predictive value, and AUC of 88%, 90%, 86%, 92%, and 0.944, respectively [10]. In their subsequent study, they investigated MMAR and QCA, as in our study, and a ratio of MMAR to MLD^4^ >6.3 predicted an FFR < 0.80 with a sensitivity, specificity, and AUC of 73%, 72%, and 0.78, respectively [23]. In contrast, Kim et al. used the fractional myocardial mass (which we refer to as MMAR) with the stem-and-crown model and QCA data and revealed that the optimal cut-off of MMAR/MLD was 28.8 g/mm, with sensitivity, specificity, positive predictive value, negative predictive value, and accuracy of 75%, 77%, 68%, 83%, and 77%, respectively [9]. These excellent results using similar indexes for predicting functional significance and its consistency support the high diagnostic accuracy of MMAR/MLD in the present study. In addition, of note, only the QCA and CCTA findings analyzed with the standard software program were used in our study.

In the subgroup analysis, a significant correlation between MMAR/MLD and FFR was demonstrated in the right coronary, left anterior descending, and left circumflex arteries. The AUCs of MMAR/MLD for FFR ≤ 0.8 in each coronary artery were in the range of 0.710–0.805, indicating that these relationships were consistent among all coronary arteries. In addition, the cut-off values were 29.5, 29.5, and 29.9 in all the culprit lesions, the culprit lesions in the right coronary artery, and the culprit lesions in the left anterior descending artery, respectively. Interestingly, these values were similar to those of the previous report that determined the cut-off value to be 28.8 g/ml using a research version of the software program with the stem-and-crown model [9], although the value in the left circumflex artery was relatively low. The consistency of the relationships and the good diagnostic performance support the usefulness of the novel index of MMAR/MLD using the standard software program in daily practice. Of further note, the AUC of MMAR/MLD for FFR ≤ 0.8 in the proximal left anterior descending artery lesions was 0.919. This high diagnostic accuracy of MMAR/MLD suggests that the index might be of particular use for assessing the proximal left anterior descending artery lesions because the lesion location has a high frequency of discordance between anatomical severity and functional severity [8].

Recently, CCTA-based FFR was developed for the noninvasive functional diagnosis of coronary artery disease and is becoming an examination tool in the daily practice [24, 25]. Similar to CCTA-based FFR, MMAR/MLD is a CCTA-based method to predict functional stenosis; however, there are some critical differences between them. MMAR can be automatically calculated with several clicks in a few minutes using the standard software program on a workstation-based system. This eliminates any extra cost or need to transfer CCTA data outside of the institution. Several new software programs for the workstation-based system are being developed but are not yet available in daily practice. The main disadvantage associated with MMAR/MLD is the use of QCA; however, the precise anatomical evaluation might be a reason for the good correlation between the MMAR/MLD and FFR.

Using the novel index of MMAR/MLD in real-world practice may allow us to skip the step of FFR measurement when determining the indication for revascularization, which will save on time and costs for the procedure. Even for patients with multivessel disease, functional assessments with FFR measurements are important and change the treatment strategy in 43% of patients [26]. However, assessing FFR in multiple lesions in multiple vessels using a pressure wire can be difficult in terms of the manipulation capability, the durability of the wire, the time for the procedure, and the cost of the drugs for hyperemia. In contrast, MMAR/MLD can be easily assessed with several clicks and a few simple calculations, even with multiple lesions, so making decisions using MMAR/MLD seems relatively easy and quite feasible in daily practice.

Several limitations associated with the present study warrant mention. First, the study population was not very large, although our study was performed at four institutions. Second, we did not compare the MMAR/MLD to the previously reported index. Third, we excluded tandem lesions and severe diffuse lesions, which were deemed to be major targets for FFR assessment in daily practice. However, issues are emerging in the determination of FFR for each stenosis in isolation and the accurate prediction of postprocedural FFR values in tandem or diffuse disease, as fluid dynamic interaction occurs between lesions during maximal hyperemia, such that the FFR of a proximal stenosis is influenced by the presence of a distal stenosis, and vice versa. In addition, a recent study revealed that constructing an interventional strategy based on resting index measurements might be promising, especially for tandem or diffuse lesions, in order to achieve a physiologically favorable result [27]. Therefore, the exclusion of tandem lesions and diffuse lesions from our study seemed to be appropriate, and an investigation using a resting index might be able to overcome issues with these lesions. However, we included 4 lesions in the infarct-related vessel, and the relationships between the MMAR/MLD and FFR in the remaining 145 lesions were not markedly different after the exclusion of those 4 lesions. This consistency supports the advantage of MMAR calculated by CCTA for estimating perfusion territories accurately, even in cases of myocardial infarction. Fourth, CCTA and QCA results are needed in order to calculate MMAR/MLD, so the use of the index is limited in patients who have undergone CCTA prior to CAG. Fifth, the prognostic implication of MMAR/MLD was not investigated, and thus a further study is needed.

## 5. Conclusions

In this study, we validated a novel index of MMAR/MLD for detecting functionally significant coronary stenosis. The MMAR/MLD is easily calculated from routine CAG and CCTA data using the standard software program and shows a significant correlation with FFR and good diagnostic performance for FFR ≤ 0.8. For the proximal left anterior descending artery lesions, in particular, the MMAR/MLD had a high diagnostic accuracy and may, therefore, be very helpful in assessing lesion severity.

## Figures and Tables

**Table 1:** Clinical demographics.

Age, years	71.0 (65.0–77.5)
Male	72 (65)
Body mass index (kg/m^2^)	23.9 (22.0–26.3)
Diagnosis	
Stable coronary disease	109 (98)
Acute coronary syndrome	2 (2)
Diabetes	49 (44)
Hypertension	83 (75)
Dyslipidemia	52 (47)
Smoking	44 (40)
Family history of coronary artery disease	29 (26)
Prior myocardial infarction	11 (10)
Prior percutaneous coronary intervention	34 (31)
Chronic kidney disease	27 (24)
Prior stroke	19 (17)
Left ventricular ejection fraction (%^*∗*^)	66 (61–71)
Hemoglobin (g/dl)	13.3 (12.5–14.3)
Creatinine (mg/dl)	0.85 (0.72–1.05)

Values are presented as the median (interquartile range) or number (%). ^*∗*^Echocardiography was not assessed in 2 patients.

**Table 2:** Quantitative coronary angiography and computed coronary angiography tomography.

	All	FFR ≤ 0.8	FFR > 0.8	*p* value
*n*	149	57	92	
Involved vessel				0.463
RCA	27 (18)	9 (16)	18 (20)	
LAD	90 (60)	38 (67)	52 (57)	
LCx	32 (21)	10 (18)	22 (24)	
Proximal lesion	54 (36)	22 (39)	32 (35)	0.768
FFR	0.82 (0.77–0.88)	0.75 (0.70–0.77)	0.87 (0.84–0.91)	<0.001
DS (%)	47 (40–56)	53 (45–65)	45 (39–54)	0.002
RD (mm)	2.65 (2.26–3.10)	2.56 (2.16–2.93)	2.75 (2.37–3.21)	0.025
MLD (mm)	1.39 (1.04–1.69)	1.21 (0.89–1.46)	1.49 (1.21–1.85)	<0.001
Length (mm)	12.0 (7.6–15.8)	11.9 (7.1–15.5)	12.3 (7.8–15.9)	0.848
MMAR (ml)	30.6 (21.9–44.3)	34.2 (23.8–54.1)	28.6 (20.6–40.2)	0.032
%MMAR	25.2 (18.1–33.5)	25.6 (18.3–33.2)	24.9 (18.0–33.6)	0.857

Values are presented as the median (interquartile range) or number (%). DS, diameter stenosis; FFR, fractional flow reserve; LAD, left anterior descending artery; LCx, left circumflex artery; MLD, minimal lumen diameter; MMAR, myocardial mass at risk; RCA, right coronary artery; RD, reference diameter.

## Data Availability

The data (tables and figures) used to support the findings of this study are included within the article.

## Abbreviations

FFR: Fractional flow reserve
MMAR: Myocardial mass at risk
QCA: Quantitative coronary angiography
CCTA: Coronary computed tomography angiography
CAG: Coronary angiography
MLD: Minimal luminal diameter
3D: 3-dimensional
MMAR/MLD: MMAR-to-MLD ratio
AUC: Area under the receiver operating characteristic curve
CI: Confidence interval.
